# A dataset of Raman spectra with quantitative labels of mixtures of nitrate, nitrite and sulphate

**DOI:** 10.1016/j.dib.2026.113319

**Published:** 2026-10-07

**Authors:** Lorenzo Luciani, Antonio Nocera, Michela Raimondi, Agnese Sbrollini, Micaela Morettini, Rossana Galassi

**Affiliations:** a Scuola di Scienze e Tecnologie, Chemistry Division, Università di Camerino, Camerino, Italy; b Dipartimento di Ingegneria dell’Informazione, Università Politecnica delle Marche, Ancona, Italy

**Keywords:** Spectroscopy, Quantification, Pollutants, Environmental monitoring, Nitrate, Nitrite, Sulphate

## Abstract

This article describes a dataset of Raman spectra acquired from aqueous solutions containing nitrate, nitrite, and sulphate ions at known concentration levels. The dataset includes 694 raw Raman spectra collected in the Stokes region from 400 to 3900 cm⁻¹, and each spectrum is associated with quantitative labels reporting the concentrations of nitrate, nitrite, and sulphate in mg/L. The data include single-analyte, binary-mixture, and ternary-mixture samples, covering a range of concentration values for the three target anions. The spectra were recorded using a micro-Raman system integrating an Olympus BXFM microscope, a 50× objective, and a Horiba iHR320 spectrometer equipped with a Horiba Syncerity CCD detector, with measurements performed under 532 nm excitation. The dataset is organized in spreadsheet files in which spectral intensity values are reported as a function of wavenumber, followed by the three quantitative concentration labels for each sample. This dataset can support the development, validation, and comparison of signal processing, chemometric, and machine learning approaches for Raman-based water analysis, including both qualitative recognition and quantitative estimation tasks in single- and multi-analyte conditions.

Specifications Table

**Table:** 

Subject	Chemistry
Specific subject area	Analytical Chemistry, Quantitative Spectroscopy
Type of data	Raw Table data
Data collection	All experiments were conducted using a micro-Raman instrumentation integrating an Olympus BXFM microscope, with the laser beam focused through a 50× objective and a diffraction grating with 600 lines/mm; the Raman spectra were obtained with a spectrometer (Horiba iHR320) in Czerny-Turner geometry equipped with a Horiba Syncerity CCD camera detector. The spectrum was collected in the Stokes region over the wavenumber range 400 cm^−1^ to 3900 cm^−1^, excluding the elastic peak with a long-pass edge filter. Water solutions with dissolved nitrate, nitrite, and sulphate anions at different concentration levels are recorded. The experiments involved single-analyte, binary, and ternary mixtures.
Data source location	Università di Camerino, Science and Technology School, Chemistry Faculty
Data accessibility	Repository name: Mendeley Data Data identification number: 10.17632/cxf3zxsc3k.3 Direct URL to data: https://data.mendeley.com/datasets/cxf3zxsc3k/3
Related research article	Nocera A, Luciani L, Ciattaglia G, Raimondi M, Burattini L, Spinsante S, et al. AI-assisted methodology for robust digital measurements by Raman spectroscopy: Quantification of inorganic pollutants in water. Measurement. 2026 Mar; 265:120429. doi:10.1016/j.measurement.2026.120429 [1]

## 1 Value of the Data

• The dataset provides both single-analyte and mixture-of-analytes spectra. Therefore, it gives both simpler cases and more compositionally complex cases to study the spectral response of pollutants in ultrapure water samples.
• The dataset provides a set of Raman spectra of pollutants in water samples with the associated quantity of the pollutants. The quantitative labels, corresponding to the concentration of nitrate, nitrite, and sulphate, provide added value to the data when compared to the landscape of Raman datasets, which focus heavily on spectral classification. An example is the RRUFF dataset [2], which provides a large-scale dataset for identifying minerals.
• Raman spectroscopy is commonly employed as a qualitative technique for the identification of substances, whereas the dataset provides data with quantity labels. The dataset supports the advancement, through development and benchmarking, of Raman quantitative detection methods. The quantitative models include low-complexity machine learning models for the task of multi-output regression of pollutant quantity in water samples.
• Potential beneficiaries of the data are chemists and spectroscopic experts because the data provide the spectra of three important pollutants present in water under laboratory conditions. Other potential beneficiaries include experts in quantitative and qualitative models for spectroscopic data.

## 2 Background

Recently, water contamination issues resulting from agricultural and industrial practices have intensified, leading to a demand for rapid and dependable analytical systems [[3], [4], [5], [6]]. In this regard, Raman spectroscopy (RS) has emerged as a significant technique for environmental monitoring, evaluating the presence of anions in potable water with minimal sample preparation [7]. Nevertheless, the quantification of contaminant concentrations poses challenges due to the weak Raman signals, inadequate sensitivity, and the impact of noise, fluorescence, and instrumentation differences.

Artificial Intelligence (AI) appears as one of the most promising solutions to improve the RS capabilities for performing automated analysis of Raman spectra and the identification of substances [1,8]. The RRUFF dataset [2] enabled research in spectral classification of solid minerals with AI-assisted qualitative identification of substances.

In contrast, quantitative methods for RS are less developed due to the lack of data [9]. This paper fills this gap by providing the quantitative labels of the dissolved pollutants associated with each spectrum. Consequently, the goal of this paper and the associated paper [1] is to promote the development of quantification methods for inorganic water pollutants through RS. Nitrate, nitrite, and sulphate ions are examples of such a class of contaminants.

## 3 Data Description

A total of 694 raw Raman spectra of anion water solutions are presented. Each Raman spectrum intensity is characterized by arbitrary unit values and wavenumbers from 400 to 3900 cm^−1^. Each spectrum is associated with a certain quantity of one or multiple nitrate, nitrite, and sulphate anions dissolved in water. The data are collected in a single tabular data file organized as shown in Fig. 1. The same data is reported in the form of an Excel file (“all_spectra_dataset.xlsx”) and a comma-separated values file (“all_spectra_dataset_CSV.csv”). The tabular data has a header in which the number corresponds to the sampled wavenumber in cm^−1^, and the last three values correspond to the quantitative data labels for the concentrations of Nitrate, Nitrite, and/or Sulphate, respectively, in mg/L. The first column of the tabular data corresponds to the row identifier number (Spectra ID), going from 0 to 693. A README.md file is included and contains a general dataset overview, the included files, a data dictionary describing what each file contains, and different sections commenting on the data headers of the included files.

**Fig. 1:**
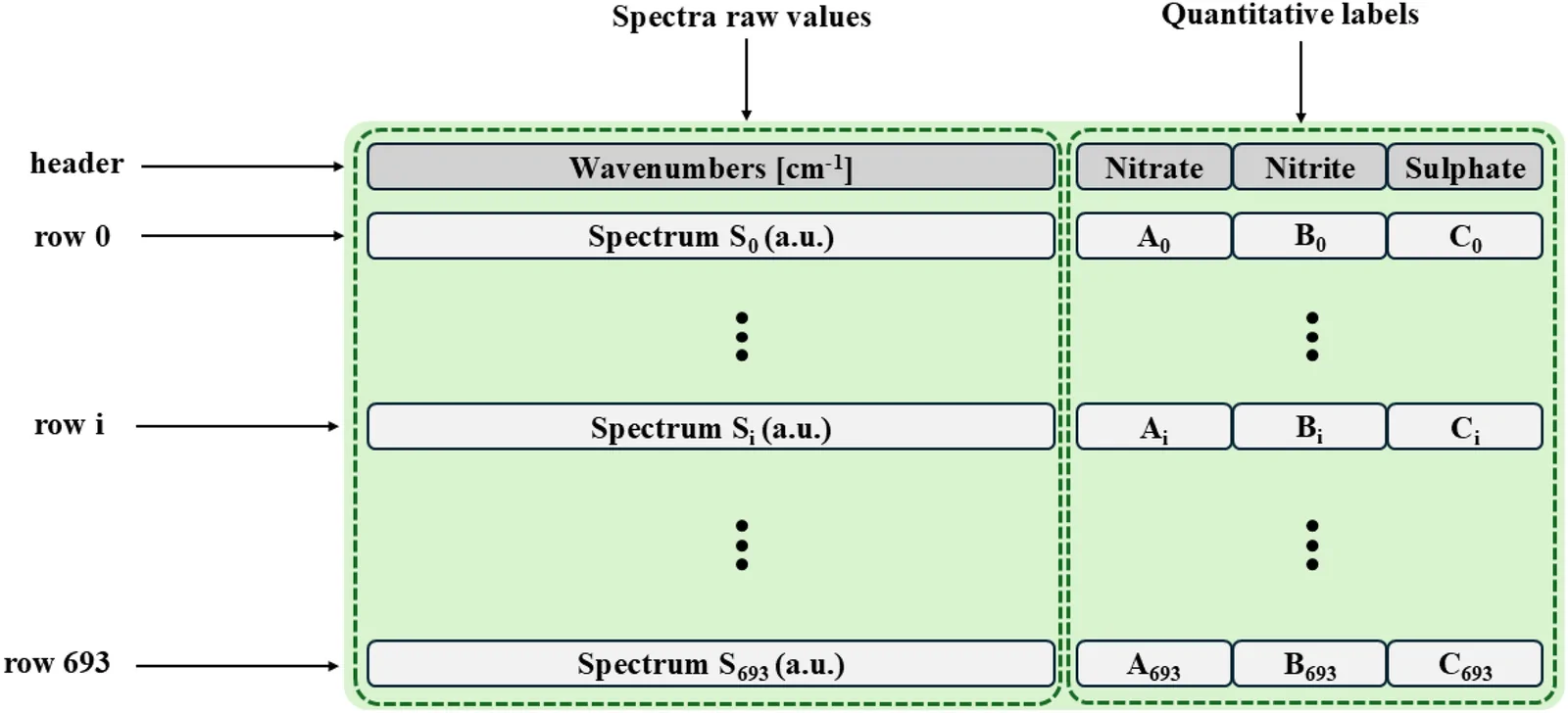
Schematic representation of the dataset with the raw spectra.

Table 1 reports a description of the number of spectra across single-analyte solutions, binary solutions, and ternary solutions, and their concentration ranges.

**Table 1:** Description of the dataset cardinality, type of solutions, and concentration ranges.

Number of spectra	Analytes	Concentration ranges
149	Nitrate	122–30667 mg/L
70	Nitrite	250–10000 mg/L
70	Sulphate	253–5072 mg/L
140	Nitrate/Nitrite	383–15333 mg/L (each)
75	Nitrate/Sulphate	422–16907 mg/L (each)
190	Nitrate/Nitrite/Sulphate	255–15333 mg/L (each)

In particular, the dataset has 70 spectra of single sulphate anion solutions, 149 spectra of single nitrate anion solutions, and 70 spectra of single nitrite anion solutions; for the binary solutions, the dataset has 140 spectra for nitrate and nitrite solutions, and 75 spectra for sulphate and nitrate solutions; for the ternary solutions, the dataset has 190 spectra.

The concentration range includes high concentration levels, selected considering the sensitivity of traditional Raman Spectroscopy. Given the weak Raman signal of the studied analytes, high concentrations are necessary to obtain a measurable deviation in Raman intensity. Consequently, the dataset supports proof-of-concept development of quantitative methods.

Table 2 shows a sample-composition table, reporting for each measured solution the analyte concentrations, the relative counterions, the mixture type (single, binary, or ternary), the concentration ratio, and the number of replicates. A full sample-composition table with all the repeated measurements for each combination of analytes is available as a comma-separated values file at the link to the dataset (“full_sample_composition_table_CSV.csv”). The full sample-composition table reports each experiment performed, with the Spectra ID referring to the main dataset tabular file (“all_spectra_dataset.xlsx” or “all_spectra_dataset_CSV.csv”), the relative concentrations of the Nitrate, Nitrite and/or Sulphate anions in mg/L, the counterions, the mixture type (single, binary or ternary), the concentration ratios between the appearing concentrations and the replicate number, representing the replicate measurement ID of that specific spectra. No specific preparation ID appears because each unique analyte concentration combination corresponds to one uniquely prepared solution. The replicate ID gives information on the replicated measurement for the same unique analyte concentration combination.

**Table 2:** Complete sample composition structure of the dataset.

Nitrate [mg/L]	Nitrite [mg/L]	Sulphate [mg/L]	Counterions	Mixture type	Concentration ratio	Number of replicates
1022	1022	0	K^+^, Na^+^	binary	1:1	10
1533	1533	0	K^+^, Na^+^	binary	1:1	10
511	511	0	K^+^, Na^+^	binary	1:1	10
15333	15333	0	K^+^, Na^+^	binary	1:1	10
3066	3066	0	K^+^, Na^+^	binary	1:1	10
7666	7666	0	K^+^, Na^+^	binary	1:1	10
3067	15333	0	K^+^, Na^+^	binary	1:5	10
383	1916	0	K^+^, Na^+^	binary	1:5	10
767	3833	0	K^+^, Na^+^	binary	1:5	10
1533	7666	0	K^+^, Na^+^	binary	1:5	10
3833	767	0	K^+^, Na^+^	binary	5:1	10
15333	3067	0	K^+^, Na^+^	binary	5:1	10
1916	383	0	K^+^, Na^+^	binary	5:1	10
7666	1533	0	K^+^, Na^+^	binary	5:1	10
676	0	3381	K^+^, 2Na^+^	binary	1:5	10
1690	0	8453	K^+^, 2Na^+^	binary	1:5	9
3381	0	16907	K^+^, 2Na^+^	binary	1:5	9
1690	0	1690	K^+^, 2Na^+^	binary	1:1	9
16907	0	16907	K^+^, 2Na^+^	binary	1:1	10
3381	0	3381	K^+^, 2Na^+^	binary	1:1	9
422	0	422	K^+^, 2Na^+^	binary	1:1	10
845	0	845	K^+^, 2Na^+^	binary	1:1	9
122	0	0	K^+^	single	1	10
1533	0	0	K^+^	single	1	10
15333	0	0	K^+^	single	1	10
306	0	0	K^+^	single	1	10
3066	0	0	K^+^	single	1	10
30667	0	0	K^+^	single	1	10
613	0	0	K^+^	single	1	10
6133	0	0	K^+^	single	1	10
1000	0	0	K^+^	single	1	10
1250	0	0	K^+^	single	1	10
250	0	0	K^+^	single	1	10
2500	0	0	K^+^	single	1	9
500	0	0	K^+^	single	1	10
5000	0	0	K^+^	single	1	10
800	0	0	K^+^	single	1	10
0	1000	0	Na^+^	single	1	10
0	10000	0	Na^+^	single	1	10
0	250	0	Na^+^	single	1	10
0	2500	0	Na^+^	single	1	10
0	500	0	Na^+^	single	1	10
0	5000	0	Na^+^	single	1	10
0	750	0	Na^+^	single	1	10
0	0	1014	2Na^+^	single	1	10
0	0	1690	2Na^+^	single	1	10
0	0	253	2Na^+^	single	1	10
0	0	2536	2Na^+^	single	1	10
0	0	507	2Na^+^	single	1	10
0	0	5072	2Na^+^	single	1	10
0	0	845	2Na^+^	single	1	10
1022	1022	1022	K^+^,3Na^+^	ternary	1:1:1	10
15333	15333	15333	K^+^,3Na^+^	ternary	1:1:1	10
1533	1533	1533	K^+^,3Na^+^	ternary	1:1:1	10
255	255	255	K^+^,3Na^+^	ternary	1:1:1	10
3066	3066	3066	K^+^,3Na^+^	ternary	1:1:1	10
511	511	511	K^+^,3Na^+^	ternary	1:1:1	10
7665	7665	7665	K^+^,3Na^+^	ternary	1:1:1	10
3067	15333	3067	K^+^,3Na^+^	ternary	1:5:1	10
383	1916	383	K^+^,3Na^+^	ternary	1:5:1	10
767	3833	767	K^+^,3Na^+^	ternary	1:5:1	10
1533	7666	1533	K^+^,3Na^+^	ternary	1:5:1	10
15333	3067	3067	K^+^,3Na^+^	ternary	5:1:1	10
1916	383	383	K^+^,3Na^+^	ternary	5:1:1	10
3833	767	767	K^+^,3Na^+^	ternary	5:1:1	10
7666	1533	1533	K^+^,3Na^+^	ternary	5:1:1	10
3067	3067	15333	K^+^,3Na^+^	ternary	1:1:5	10
383	383	1916	K^+^,3Na^+^	ternary	1:1:5	10
767	767	3833	K^+^,3Na^+^	ternary	1:1:5	10
1533	1533	7666	K^+^,3Na^+^	ternary	1:1:5	10

From Table 2, it is possible to observe that not all tested concentration ratios have 10 repeated measurements. The missing replicate measurements are attributable to technical issues during data collection, specifically acquisition failures or software-related failures during data upload. Consequently, for some concentration conditions, only nine valid spectra were available instead of the intended ten replicates.

An example of the obtained raw spectra for the multi-analyte cases, including Nitrate, is reported in Fig. 2.

**Fig. 2:**
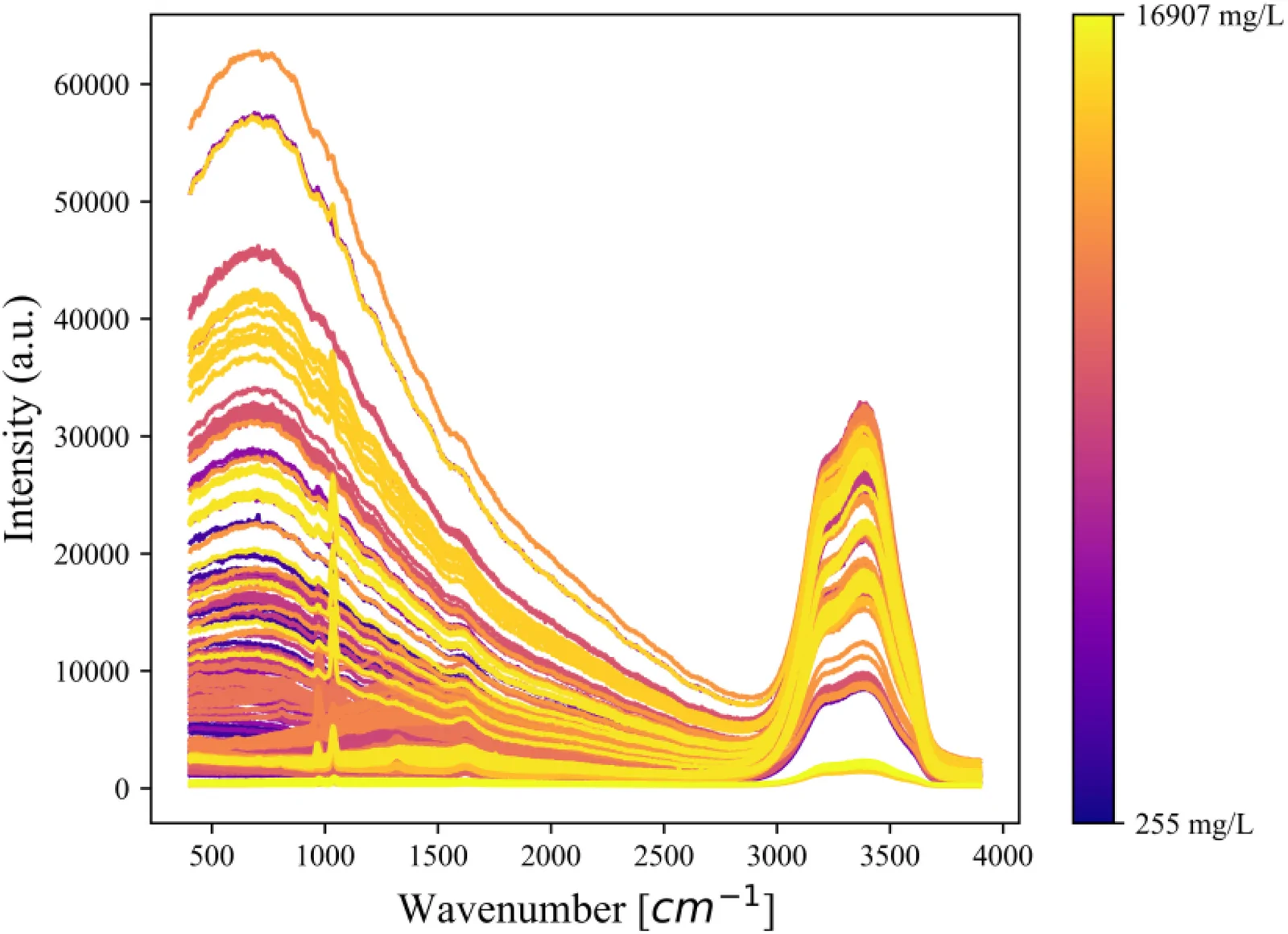
Representative raw Raman spectra of multi-analyte aqueous samples containing nitrate, with color scale indicating nitrate concentration.

The dataset can be employed to develop low-complexity machine learning models, such as linear regressors, support vector regressors, and decision trees. A suggested split, employed in the associated paper [1], is to use the single-analyte cases as the development set for training and validation purposes, splitting the examples by combination of concentrations to remove potential data leakage. The remaining binary and ternary cases can be used as unseen test examples. This development and test split enables to demonstrate a generalization of the quantification methods from simple single-analyte cases to a slightly more complex scenario of a mixture of analytes.

The next section gives specific details about what concentrations appear in the dataset and how they are obtained.

## 4 Experimental Design, Materials and Methods

The Raman platform consisted of a micro-Raman system equipped with two Olympus microscope configurations, allowing the optical arrangement to be adapted according to sample type and measurement needs. Depending on the experiment, excitation could be selected from 532, 633, or 785 nm laser sources. For these experiments, the measurements were carried out under 532 nm excitation at a constant output power of 20 mW, using the BXFM microscope and a 50× objective for beam focusing.

Fiber-coupled laser configuration enabled focusing a beam in the order of 2 to 5 µm, while the scattered signal was collected in backscattering geometry and analyzed with a Horiba iHR320 Czerny–Turner spectrometer coupled to a Horiba Syncerity CCD detector. A long-pass edge filter was included in the collection path to suppress elastically scattered light, and a 600 grooves/mm grating was chosen to balance spectral range and resolution. A schematic representation of the instrumental configuration is presented in Fig. 3.

**Fig. 3:**
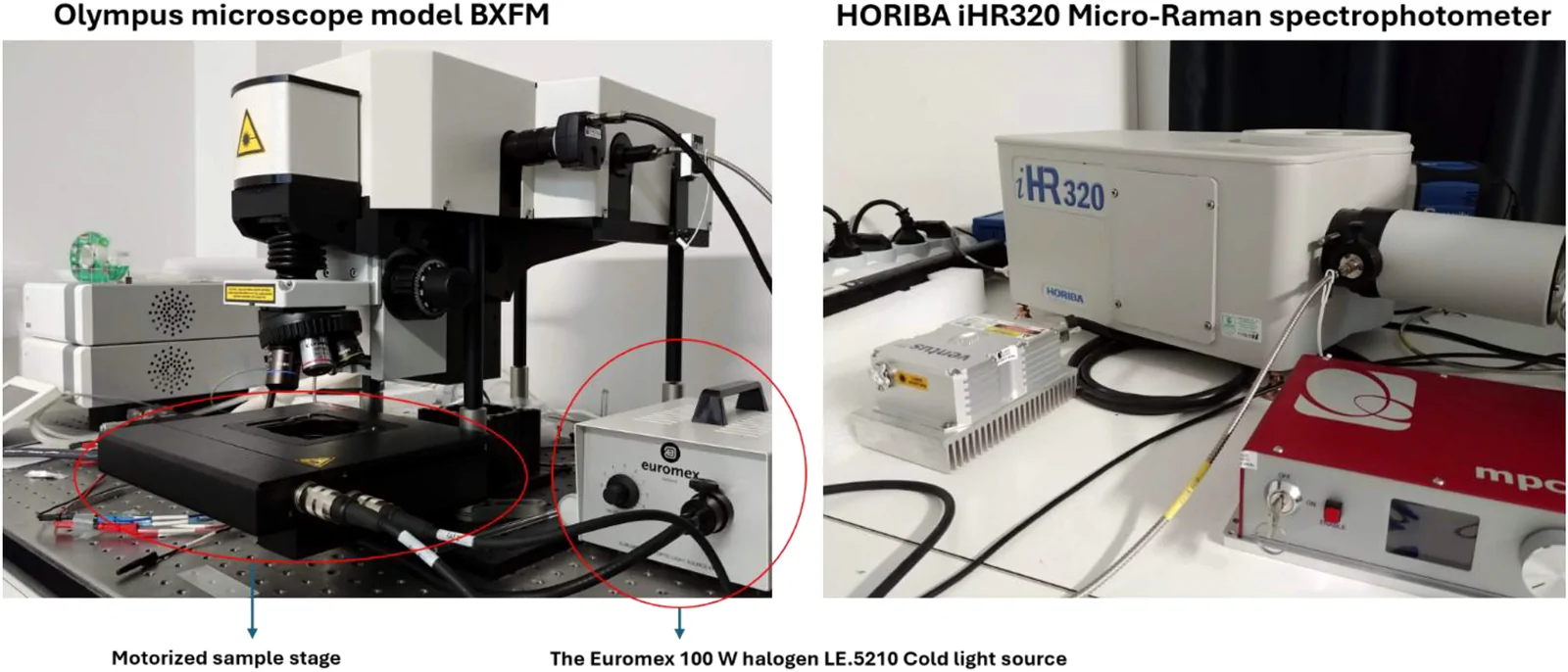
Layout of the Horiba iHR320 Micro-Raman spectrophotometer, and of the Olympus microscope model BXFM and its components.

### 4.1 Detailed Solution Preparation Procedures

Spectral measurements were performed on liquid samples transferred into open Wheaton vials, with the optical focus positioned approximately 800 µm beneath the solution. The analyte solutions were obtained from stock preparations made with ultrapure Milli-Q water in Wheaton glass vials. Commercial sodium or potassium anhydrous salts of analytical purity greater than 99% were used as received, after storage in a desiccator to reduce moisture uptake. From the initial stock solutions, further samples were prepared by volumetric dilution to cover the concentration interval required for calibration and testing. This dilution procedure ensured controlled concentration levels across the experimental dataset. The detailed procedure used to prepare all the studied solutions is reported in the next sections. The reported concentrations were calculated from the gravimetric and volumetric preparation procedures and were not independently confirmed using a separate analytical method; therefore, they are referred to as nominal concentrations.

Raman spectra were acquired using a 198 µm (0.198 mm) front entrance slit, a Horiba Syncerity CCD detector with 1024 pixels, and a 600 grooves/mm diffraction grating, corresponding to an estimated spectral resolution of approximately 0.94 nm under the employed acquisition conditions. Before the spectral acquisition process, the laser wavelength and diffraction grating were chosen based on the experimental configuration, and the spectral calibration was validated with a high-purity crystalline silicon reference sample. Specifically, the characteristic Raman band of silicon, located at approximately 520.7 cm⁻¹, was employed as the reference for the calibration procedure. An automatic calibration procedure was then carried out using the autocalibration feature embedded in LabSpec6 software version 6.4.3.633. For every single component, binary and ternary solutions at a specified concentration, as detailed in the subsequent section, up to 10 spectra were acquired by averaging 10 scans with an integration time of 5 seconds (50 seconds in total per spectrum). This established and optimized procedure was chosen to facilitate the collection of replicate data for statistical evaluation. All spectral acquisition and data processing were performed using LabSpec6 software version 6.4.3.633. This strategy was adopted to improve repeatability and to limit the effect of random fluctuations on the recorded Raman response. The obtained spectra have a sampling interval of about 4 cm^−1^. The wavenumber axis is available in the dataset in the first row; therefore, the precise interval between consecutive sampling points can be obtained from the first row of the published dataset.

After acquisition, spectra from single-component samples as well as binary and ternary mixtures were inspected by applying a spline-based baseline treatment within a peak-analysis routine to check signal quality and internal consistency. The data discussed in the study correspond to the raw spectra. The detailed procedures associated with each experimental case are presented separately in the next methodological sections.

### 4.2 Preparation of Single Analyte Solutions of Nitrate, Nitrite, and Sulphate Solution

All the pure salts were weighed directly into the 5 mL calibrated flasks by using a Mettler Toledo AT201 Analytical Balance (uncertainty = ± 0.01 mg). For the mixture of analyte solutions, the indicated mixture ratios refer to nominal mass/volume concentration ratios expressed in mg/L.

#### 4.2.1 Nitrite Anion Solutions

75.00 mg of pure NaNO_2_ salt was dissolved in ultrapure Milli-Q® water in a 5 mL (± 0.025 mL) calibrated volumetric flask to obtain a stock solution at a nominal concentration of 10000 mg/L of NO_2_^−^ anion. Subsequently, the stock solution was diluted in standard Wheaton glassware using a Gilson Micropipette to obtain solutions at the following nominal concentrations:

(a) 5000 mg/L
(b) 2500 mg/L
(c) 1000 mg/L
(d) 750 mg/L
(e) 500 mg/L
(f) 250 mg/L

#### 4.2.2 Sulphate Anion Solutions

37.51 mg of pure Na_2_SO_4_ was dissolved in ultrapure Milli-Q® water in a 5 mL (± 0.025 mL) calibrated volumetric flask to obtain a stock solution at a nominal concentration of 5072 mg/L of SO_4_^2−^ anion. Subsequently, the stock solution was diluted in standard Wheaton glassware using a Gilson Micropipette to obtain solutions at the following nominal concentrations:

(a) 2536 mg/L
(b) 1690 mg/L
(c) 1014 mg/L
(d) 845 mg/L
(e) 507 mg/L
(f) 253 mg/L

#### 4.2.3 Nitrate Anion Solutions

250.13 mg of pure KNO_3_ was dissolved in ultrapure Milli-Q®water in a 5 mL (± 0.025 mL) calibrated volumetric flask to obtain a stock solution at a nominal concentration of 30667 mg/L of NO_3_^−^ anion. Subsequently, the stock solution was diluted in standard Wheaton glassware using a Gilson Micropipette to obtain solutions at the following nominal concentrations:

(a) 15333 mg/L
(b) 6133 mg/L
(c) 3066 mg/L
(d) 1533 mg/L
(e) 613 mg/L
(f) 306 mg/L
(g) 122 mg/L

Moreover, a stock solution at a nominal concentration of 5000 mg/L of NO_3_^−^ anion is generated by dissolving 40.77 mg of pure KNO_3_ in a 5 mL (± 0.025 mL) calibrated volumetric flask and diluted to obtain the following nominal concentrations:

(a) 2500 mg/L
(b) 1250 mg/L
(c) 1000 mg/L
(d) 800 mg/L
(e) 500 mg/L
(f) 250 mg/L

### 4.3 Preparation of Binary Mixture Solutions of Nitrate, Nitrite and Sulphate Solution

The binary mixture solutions of nitrate/nitrite and nitrate/sulphate are included, whereas the mixture solutions of nitrite/sulphate are absent.

#### 4.3.1 Binary Mixture in 1:1 Ratio of Nitrate and Nitrite Anions

125.01 mg of KNO_3_ and 114.98 mg of NaNO_2_ were dissolved in ultrapure Milli-Q®water in a 5 mL (± 0.025 mL) calibrated volumetric flask to obtain nitrate (NO_3_^−^) and nitrite (NO_2_^−^), stock solution in a concentration ratio of 1:1 at a nominal concentration of 15333 mg/L. Subsequently, the stock solution was diluted in standard Wheaton glassware using a Gilson Micropipette to obtain solutions at the following nominal concentrations:

(a) 7666 mg/L
(b) 3066 mg/L
(c) 1533 mg/L
(d) 1022 mg/L
(e) 511 mg/L

#### 4.3.2 Binary Mixture in 5:1 Ratio of Nitrate and Nitrite Anions

125.01 mg of KNO_3_ and 23.00 mg of NaNO_2_ were dissolved in ultrapure Milli-Q®water in a 5 mL (± 0.025 mL) calibrated volumetric flask to obtain nitrate (NO_3_^−^), nitrite (NO_2_^−^) stock solution in a 5:1 ratio at nominal concentrations of 15333 mg/L and 3067 mg/L, respectively. Subsequently, the stock solution was diluted in standard Wheaton glassware using a Gilson Micropipette to obtain solutions at the following nominal concentrations:

(a) 7666 mg/L of nitrate; 1533 mg/L of nitrite
(b) 3833 mg/L of nitrate; 767 mg/L of nitrite
(c) 1916 mg/L of nitrate; 383 mg/L of nitrite

#### 4.3.3 Binary Mixture in 5:1 Ratio of Nitrite and Nitrate Anions

25.01 mg of KNO_3_ and 115.00 mg of NaNO_2_ were dissolved in ultrapure Milli-Q®water in a 5 mL (± 0.025 mL) calibrated volumetric flask to obtain nitrite (NO_2_^−^) and nitrate (NO_3_^−^), stock solution in a 5:1 ratio at nominal concentrations of 15333 mg/L (stock solution) and 3067 mg/L, respectively. Subsequently, the stock solution was diluted in standard Wheaton glassware using a Gilson Micropipette to obtain solutions at the following nominal concentrations:

a) 7666 mg/L of nitrite; 1533 mg/L of nitrate
b) 3833 mg/L of nitrite; 767 mg/L of nitrate
c) 1916 mg/L of nitrite; 383 mg/L of nitrate

#### 4.3.4 Binary Mixture in 1:1 Ratio of Sulphate and Nitrate Anions

125.01 mg of Na_2_SO_4_ and 137.90 mg of KNO_3_ were dissolved in ultrapure Milli-Q®water in a 5 mL (± 0.025 mL) calibrated volumetric flask to obtain sulphate (SO_4_^2−^) and nitrate (NO_3_^−^) stock solution in a 1:1 ratio at a nominal concentration of 16907 mg/L. Subsequently, the stock solution was diluted in standard Wheaton glassware using a Gilson Micropipette to obtain solutions at the following nominal concentrations:

(a) 3381 mg/L
(b) 1690 mg/L
(c) 845 mg/L
(d) 422 mg/L

#### 4.3.5 Binary Mixture in 5:1 Ratio of Sulphate and Nitrate Anions

125.01 mg of pure Na_2_SO_4_ and 27.57 mg of KNO_3_ were dissolved in ultrapure Milli-Q®water in a 5 mL (± 0.025 mL) calibrated volumetric flask to obtain sulphate (SO_4_^2−^) and nitrate (NO_3_^−^) stock solution in a 5:1 ratio at nominal concentrations of 16907 mg/L and 3381 mg/L, respectively. Subsequently, the stock solution was diluted in standard Wheaton glassware using a Gilson Micropipette to obtain solutions at the following nominal concentrations:

(a) 8453 mg/L of sulphate; 1690 mg/L of nitrate
(b) 3381 mg/L of sulphate; 676 mg/L of nitrate

### 4.4 Preparation of Ternary Mixture Solution of Nitrate, Nitrite and Sulphate Solution

#### 4.4.1 Ternary Mixture in 1:1:1 Ratio

125.01 mg of KNO_3_, 115.00 mg of NaNO_2_, and 113.40 mg of Na_2_SO_4_ were dissolved in ultrapure Milli-Q®water in a 5 mL (± 0.025 mL) calibrated volumetric flask to obtain nitrate (NO_3_^−^), nitrite (NO_2_^−^), and sulphate (SO_4_^2−^) stock solution at a nominal concentration of 15333 mg/L (stock solution). Subsequently, the stock solution was diluted in standard Wheaton glassware using a Gilson Micropipette to obtain solutions at the following nominal concentrations:

(a) 7665 mg/L
(b) 3066 mg/L
(c) 1533 mg/L
(d) 1022 mg/L
(e) 511 mg/L
(f) 255 mg/L

#### 4.4.2 Ternary Mixture of Sulphate, Nitrate and Nitrite Solution in 5:1:1 Ratio

113.40 mg of Na_2_SO_4_, 25.01 mg of KNO_3_ and 23.00 mg of NaNO_2_ were dissolved in ultrapure Milli-Q®water in a 5 mL (± 0.025 mL) calibrated volumetric flask to obtain sulphate (SO_4_^2−^), nitrate (NO_3_^−^) and nitrite (NO_2_^−^), stock solution in a mass/volume concentration ratio in mg/L of 5:1:1 at nominal concentrations of 15333 mg/L for sulphate and 3067 mg/L for nitrate and nitrite, respectively. Subsequently, the stock solution was diluted in standard Wheaton glassware using a Gilson Micropipette to obtain solutions at the following nominal concentrations:

(a) 7666 mg/L of sulphate; 1533 mg/L of nitrate and nitrite
(b) 3833 mg/L of sulphate; 767 mg/L of nitrate and nitrite
(c) 1916 mg/L of sulphate; 383 mg/L of nitrate and nitrite

#### 4.4.3 Ternary Mixture of Nitrate, Nitrite and Sulphate Solution in 5:1:1 Ratio

22.67 mg of Na_2_SO_4_, 125.01 mg of KNO_3_ and 23.00 mg of NaNO_2_ were dissolved in ultrapure Milli-Q®water in a 5 mL (± 0.025 mL) calibrated volumetric flask to obtain nitrate (NO_3_^−^), nitrite (NO_2_^−^), and sulphate (SO_4_^2−^) stock solution in a mass/volume concentration ratio in mg/L of 5:1:1 at nominal concentrations of 15333 mg/L for nitrate and 3067 mg/L for nitrite and sulphate, respectively. Subsequently, the stock solution was diluted in standard Wheaton glassware using a Gilson Micropipette to obtain solutions at the following nominal concentrations:

(a) 7666 mg/L of nitrate; 1533 mg/L of nitrite and sulphate
(b) 3833 mg/L of nitrate; 767 mg/L of nitrite and sulphate
(c) 1916 mg/L of nitrate; 383 mg/L of nitrite and sulphate

#### 4.4.4 Ternary Mixture of Nitrite, Nitrate and Sulphate Solution in 5:1:1 Ratio

22.67 mg of Na_2_SO_4_, 25.01 mg KNO_3_ and 115.00 mg of NaNO_2_ were dissolved in ultrapure Milli-Q®water in a 5 mL (± 0.025 mL) calibrated volumetric flask to obtain nitrite (NO_2_^−^), nitrate (NO_3_^−^), and sulphate (SO_4_^2−^) stock solution in a mass/volume concentration ratio in mg/L of 5:1:1 at nominal concentrations of 15333 mg/L for nitrite and 3067 mg/L for nitrate and sulphate, respectively. Subsequently, the stock solution was diluted in standard Wheaton glassware using a Gilson Micropipette to obtain solutions at the following nominal concentrations:

(a) 7666 mg/L of nitrite; 1533 mg/L of nitrate and sulphate
(b) 3833 mg/L of nitrite; 767 mg/L of nitrate and sulphate
(c) 1916 mg/L of nitrite; 383 mg/L of nitrate and sulphate

## Limitations

The data described in this article may be limited in sample size, making it difficult to develop a deep learning architecture. All recordings were performed in a laboratory and therefore may not be representative of real-world aqueous matrices due to the absence of real water interferents.

Additionally, the concentrations represented in this dataset were selected to generate measurable concentration-dependent responses using conventional Raman spectroscopy and to support proof-of-concept development of quantitative methods.

Regarding the technical design, the recordings were performed with a single instrument and a single excitation wavelength. The measurements were repeated up to 10 times for the same single preparation. The employed mixture ratios are restricted to only 1 to 1 ratio and 1 to 5 ratios. The mixture solution of nitrite and sulphate is absent.

## Ethics Statement

The authors have read and follow the ethical requirements for publication in Data in Brief and confirm that the current work does not involve human subjects, animal experiments, or any data collected from social media platforms.

## CRediT Author Statement

**Lorenzo Luciani**: Writing-Original Draft preparation; Methodology; Validation; Investigation. **Antonio Nocera:** Writing- Reviewing and Editing; Data Curation; Visualization. **Michela Raimondi**: Writing- Reviewing and Editing. **Agnese Sbrollini**: Writing- Reviewing and Editing. **Micaela Morettini**: Writing- Reviewing and Editing. **Rossana Galassi**: Writing-Original Draft preparation; Conceptualization; Supervision; Project administration; Funding acquisition.

## Data Availability

Mendeley DataA Dataset of Raman Spectra with Quantitative Labels of Nitrate, Nitrite and Sulphate for Application of Artificial Intelligence to Water Quality Sensing (Original data).
